# Apheresis Platelet Rich-Plasma for Regenerative Medicine: An In Vitro Study on Osteogenic Potential

**DOI:** 10.3390/ijms22168764

**Published:** 2021-08-16

**Authors:** Stefano Pulcini, Lucia Merolle, Chiara Marraccini, Eleonora Quartieri, Daniele Mori, Davide Schiroli, Pamela Berni, Barbara Iotti, Erminia Di Bartolomeo, Roberto Baricchi, Roberto Sala, Thelma A. Pertinhez

**Affiliations:** 1 Transfusion Medicine Unit, Azienda USL-IRCCS di Reggio Emilia, 42123 Reggio Emilia, Italy; Stefano.Pulcini@ausl.re.it (S.P.); Eleonora.Quartieri@unipr.it (E.Q.); davide.schiroli@ausl.re.it (D.S.); pamela.berni@ausl.re.it (P.B.); barbara.iotti@ausl.re.it (B.I.); erminia.dibartolomeo@ausl.re.it (E.D.B.); Roberto.Baricchi@ausl.re.it (R.B.); thelma.pertinhez@unipr.it (T.A.P.); 2 Department of Medicine and Surgery, University of Parma, 43125 Parma, Italy; daniele.mori@unipr.it (D.M.); roberto.sala@unipr.it (R.S.)

**Keywords:** platelet-rich plasma, hemoderivatives, growth factors, mineralization, gene expression

## Abstract

**Background**: Platelet-Rich Plasma (PRP) induces bone regeneration; however, there is low evidence supporting its efficacy in bone healing. The lack of a standardized protocol of administration represents the main obstacle to its use in the clinical routine for bone defects’ treatment. The purpose of this study was to characterize PRP and elucidate its osteogenic potential. **Methods**: Platelet count, fibrinogen levels, and growth factors concentration were measured in PRP obtained by four apheresis procedures. HOB-01-C1, a pre-osteocytic cell line, was used to examine the effects of different PRP dilutions (from 1% to 50%) on cell viability, growth, and differentiation. Gene expression of RUNX2, PHEX, COL1A1, and OCN was also assayed. **Results**: PRP showed a mean 4.6-fold increase of platelets amount compared to whole blood. Among the 36 proteins evaluated, we found the highest concentrations for PDGF isoforms, EGF, TGF-β and VEGF-D. PDGF-AA positively correlated with platelet counts. In three of the four tested units, 25% PRP induced a growth rate comparable to the positive control (10% FBS); whereas, for all the tested units, 10% PRP treatment sustained differentiation. **Conclusions**: This study showed that PRP from apheresis stimulates proliferation and differentiation of pre-osteocyte cells through the release of growth factors from platelets.

## 1. Introduction

Bone healing and remodeling are regulated by a variety of hormones and local growth factors that act on osteoblasts to promote bone tissue regeneration [1,2]. Osteoblast differentiation comprises four cellular stages (preosteoblast, mature osteoblast, pre-osteocyte, mature osteocyte) [3], each characterized by the expression of distinct protein markers and morphological features. Osteocytes, which represent 90% of the adult bone cell population, are former osteoblasts surrounded by unmineralized matrix (osteoid), that carry out the bone formation and resorption [4]. Osteoblasts are specialized, terminally differentiated cells arising from mesenchymal precursors [5] that respond to a variety of growth factors (GFs) and signalling molecules. These mediators act by binding to cell surface receptors and activate cellular responses, such as proliferation or differentiation, playing an important role in bone formation [6,7,8].

Platelet-rich plasma (PRP) is a biological substance containing a concentration of supraphysiologic platelets which has been recognized to promote osteoblasts’ proliferation, chemotaxis, differentiation and angiogenesis [8,9,10,11,12,13,14]. PRP injections or its topical use have been proposed to ameliorate bone repair mechanisms [9,12,13]. The platelet-derived growth factor (PDGF) contained in PRP seems to be responsible for the chemotactic and mitogenic effects on osteoblasts [15]. Additionally, the epidermal growth factor (EGF), which is particularly abundant in PRP, has been reported to stimulate the osteogenic potential and has even been proposed in stem cell-based therapy for bone tissue engineering applications [16].

The rationale of using PRP for bone healing and remodeling relies on the synergistic activity of GFs with other chemoattractant molecules released by the platelets’ α-granules. PRP seems to mediate the early steps of bone repair through an osteopromotive mechanism but is unable to sustain the process over time [17]. GFs availability, indeed, can be affected by the presence of fibrinogen which, aside from forming fibrin aggregates, plays a key role in preventing GFs clearance through their binding to its heparin-binding domain [18,19,20].

Despite the evidence, however, PRPs’ actual efficacy in bone healing, as well as their exact GFs composition, are still matter of discussion [21,22]. The lack of standardized dosages, the individual donor variability and the heterogeneity of manipulation procedures can lead to differences in PRP composition and, eventually, to an unpredictable treatment outcome [23,24,25,26].

The aim of our study is to establish the minimum effective concentration of apheresis PRP which is suitable for promoting osteogenic proliferation and differentiation. To reach this objective, PRP has been characterized in terms of: platelet count; fibrinogen and GFs content; effect on in vitro proliferation and differentiation of osteoblast cells. PRP units, obtained by apheresis from four donors, were characterized and tested at different concentrations on HOB-01-C1 [27,28] cell cultures, a pre-osteocytic cell-line, to evaluate their effects on cell viability, mineralization, and differentiation markers over time.

Our results will contribute to shedding light on PRP bioactive molecules’ composition and, most importantly, to develop standardized protocols.

## 2. Results

### 2.1. Study Population

Four donors were recruited for the apheresis procedure: their characteristics (sex, age, weight, blood group, blood pressure, blood count, etc.) are reported in the Table A1.

### 2.2. Platelet Count

Platelet (PLTs) count was assessed in both donors’ whole blood and PRP units (Table 1). The mean PLTs content in PRP samples was 4.6-fold higher than in whole blood (1223.3 ± 170.8 × 10^3^/μL vs. 264 ± 41.1 × 10^3^/μL). PRP Samples 2 and 3 showed the strongest enrichment, with a 5.4-fold higher PLTs count (Table 1).

### 2.3. Fibrinogen and Growth Factors Quantification

**Figure 1:**
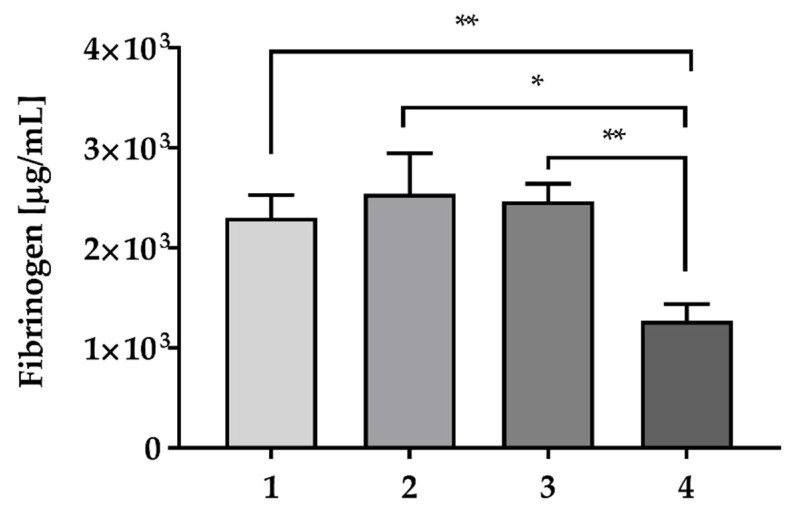
Fibrinogen concentration (μg/mL) in the PRP samples. Data are expressed as means ± SD, analysis was performed in triplicate. (* *p* < 0.05, ** *p* < 0.01).

Fibrinogen levels, measured in the PRP units by means of ELISA, are reported in Figure 1: Sample 4 showed the lowest fibrinogen content (1272.73 ± 167.28 μg/mL, * *p* < 0.05, ** *p* < 0.01), compared to Sample 1 (2303.18 ± 224.23 μg/mL), Sample 2 (2540.95 ± 404.42 μg/mL) and Sample 3 (2464.10 ± 176.99 μg/mL).

**Figure 2:**
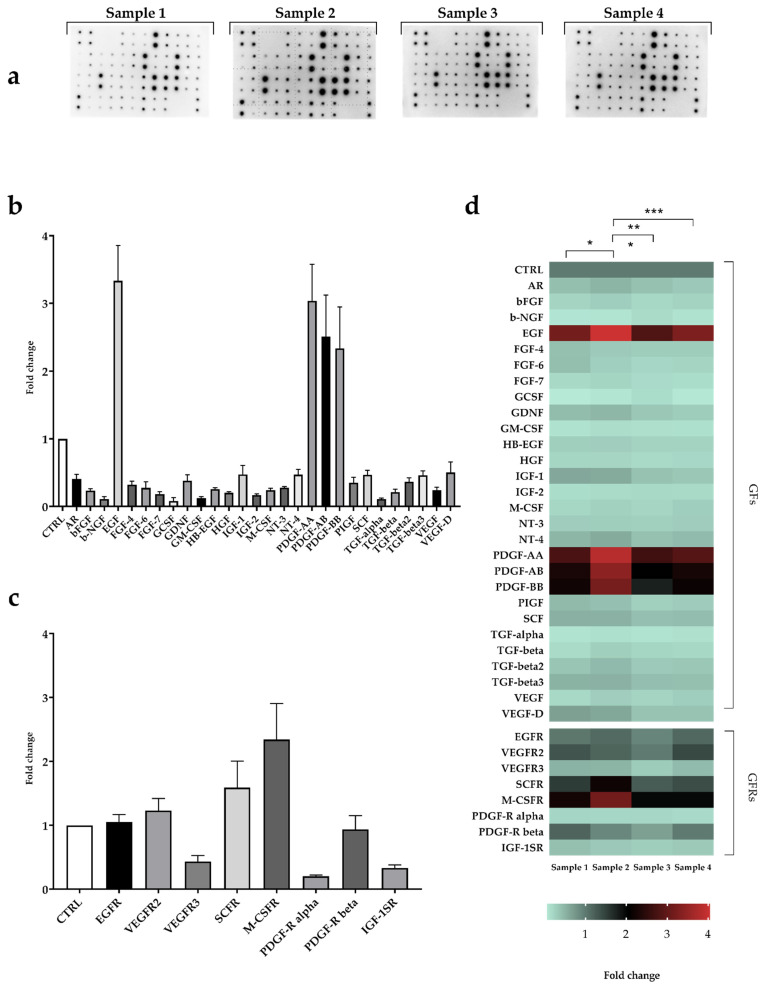
GFs and GFRs protein array. (**a**) chemiluminescence detection images of the Human Growth Factors Antibody Array membranes. Reference control (CTRL) was preloaded on the membrane by Manufacturer; (**b**) GFs and (**c**) GFRs densitometric data, expressed as mean fold change vs. control ± SD; (**d**) heatmap of the GFs and GFRs content of each PRP sample (analysis was performed in triplicate, * *p* < 0.05, ** *p* < 0.01, *** *p* < 0.001).

Growth factors released by the PRP samples were assessed exploiting a 36-proteins panel Human Growth Factors Antibody Array (Figure 2), which included 28 GFs and 8 GFs receptors (GFRs) (see Materials and Methods for details). Among the GFs (Figure 2b), EGF (3.33 ± 0.52), PDGF-AA (3.03 ± 0.53), PDGF-AB (2.50 ± 0.61), and PDGF-BB (2.33 ± 0.61) showed the highest densitometric values. GFRs intensities, instead, were higher for MCFS-R (2.34 ± 0.56) and SCFR (1.59 ± 0.42).

Despite the trend of GFs levels was similar for all the 4 PRP (Figure 2d), their relative content was significantly different. Sample 2 showed the highest protein content compared to the other PRP samples (* *p* < 0.05, *** *p* < 0.001).

Among the most represented growth factors that emerged from the protein arrays, an ELISA assay was performed to quantify PDGF-AA, -BB, -AB, and EGF. As expected, Sample 2 showed the highest concentration of all the PDGF isoforms tested, and particularly of PDGF-AA (1694.25 ± 311.23 ng/mL, Table 2). Similar results were obtained for PDGF-AB (Table 2), although PDGF-BB release was higher for Sample 2 compared to Sample 1 and Sample 4 but not compared to Sample 3. Sample 4 showed the lowest amount of PDGF-BB.

Concerning EGF, the highest concentration was found in Sample 1 (Table 2).

**Figure A1:**
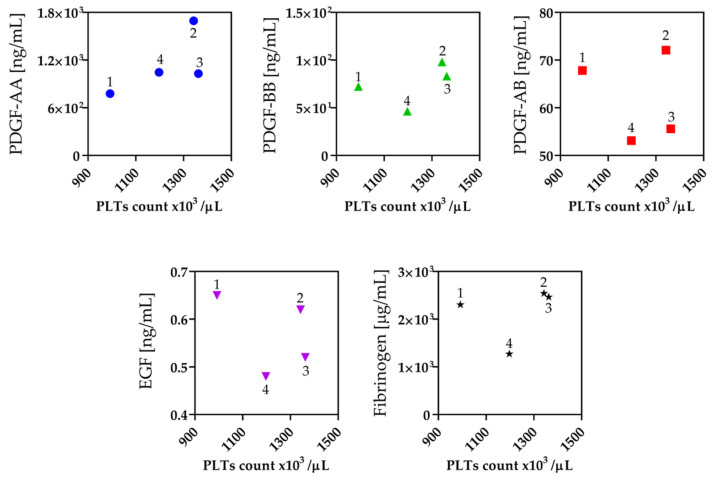
Ratio between the PLTs count and the concentration of GFs [ng/mL] or Fibrinogen [μg/mL] released by the PLTs contained in PRP samples.

Figure A1 reports the correlation graph between the PLTs count and the concentration of GFs and Fibrinogen present in PRP samples: here, the significantly high concentration of PDGF isoforms measured for Sample 2 reflects its high PLTs content. Despite having a comparable PLTs count, instead, Sample 3 showed the lowest amount of GFs, and particularly PDGF-AA and -AB. Spearman’s nonparametric test correlation revealed a positive, but not significant, correlation between PLTs and PDGF-AA and PDGF-BB (R = 0.6 and R = 0.4, respectively). 

### 2.4. In Vitro Evaluation of Osteogenic Potential

**Figure A2:**
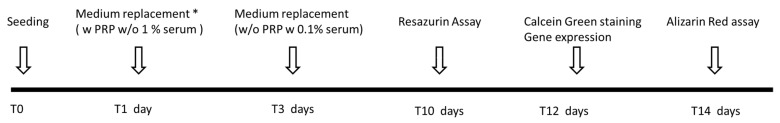
Timetable of in vitro experiments. * Details on culture media composition are reported in Section 4.

To assess the ability of PRP to stimulate proliferation and induce osteoblast-to-osteocyte transition, various dilutions (*v/v*) have been tested (from 1% to 50% final concentration) to treat the HOB-01-C1 human pre-osteocytic cell line, time-table of in vitro experiments in reported in Figure A2. Table 3 reports the GFs concentrations (ng/mL) in the culture medium corresponding to each PRP dilution.

#### 2.4.1. Cell Proliferation

**Figure 3:**
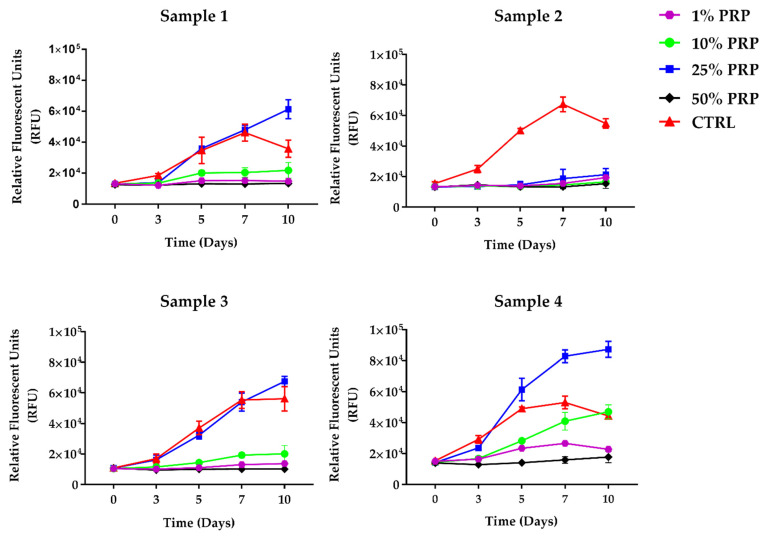
Growth curves of HOB-01-C1 cells treated with different PRP dilutions, obtained by Resazurin assay: cells seeded in complete medium containing 10% HSAB (Positive control, red curve); 1% PRP (magenta); 10% PRP (green); 25% PRP (blue), and 50% PRP (black). The *X*-axis reports the days of culture, whereas the *Y*-axis reports the relative fluorescence units (RFU). Data are expressed as mean ± SD of three independent experiments.

Resazurin-based assay was performed to evaluate HOB-01-C1 proliferation (Figure 3). After 24 h from seeding (T0), cells were treated with different PRP dilutions for 3 days; next, PRP was removed and replaced with growth medium supplemented with 0.1% human serum AB (HSAB). Resazurin assay was performed at T0, and then every two or three days after PRP removal for ten days (Timetable is reported in Figure A2).

Growth curves reported in Figure 3 suggest that HOB-01-C1 cells respond to PRP stimulus as a function of dilution, with the only exception of Sample 2 (which did not induce proliferation for any of the tested dilutions). For the other PRP, 25% dilution was sufficient to sustain cell proliferation, with growth curves approaching the positive control (Figure 3). For Sample 4, the effect on cell proliferation was even stronger than positive control. The growth pattern fits a Sigmoidal Boltzmann curve during the first 10 days of culture (Sample 1: r^2^ = 0.96; Sample 2: r^2^ = 0.43; Sample 3: r^2^ = 0.97; Sample 4: r^2^ = 0.98; CTRL 1: r^2^ = 0.81; CTRL 2: r^2^ = 0.94; CTRL 3: r^2^ = 0.95; CTRL 4: r^2^ = 0.94). Interestingly, the 50% dilution did not stimulate cell proliferation for any of the PRP tested. On the other hand, the 1% and 10% dilutions exhibited an intermediate behavior.

#### 2.4.2. Mineralized Bone Matrix Deposition

Since osteogenic differentiation leads to the deposition of calcium in the extracellular matrix, we exploited the Alizarin Red S staining to assess calcium deposition induced by PRP treatment.

HOB-01-C1 cells were cultured with different PRP dilutions for 3 days, as previously discussed. Then, PRP was removed and replaced with the osteogenic medium at 0.1% HSAB (see Materials and Methods for details). Alizarin Red S quantification was assessed after 11 days from PRP removal (T14); the timetable is reported in Figure A2.

**Figure 4:**
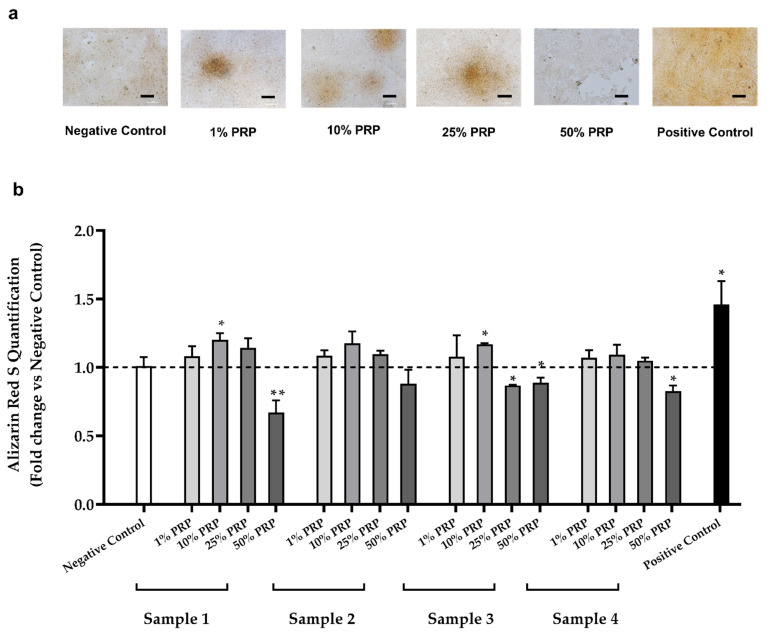
Calcium deposition in the extracellular matrix induced by PRP treatment. (**a**) Optical microscopy images of HOB-01-C1 cells treated with different PRP dilutions (1–50%) and stained at T14 with Alizarin Red S (10×) Scale bar 100 μm; (**b**) Alizarin Red S staining quantification with different PRP dilutions at T14. Data are reported as fold change vs. negative control and expressed as mean ± SD of three independent experiments. Statistical analysis was performed by one-way ANOVA test (* *p* < 0.05; ** *p* < 0.01).

As shown in Figure 4, treatment with PRP samples induced a greater calcium deposition on the extracellular matrix compared to the negative control. The strongest mineralization effect was obtained with the 10% dilution, and particularly with Samples 1 and 3 (* *p* < 0.05, Figure 4b), but calcium deposition was evident even with 1% and 25% PRP (Figure 4a). Treatment with 50% PRP did not allow cells to reach the confluence and to form bone nodules, resulting in a discontinuous extracellular matrix (Figure 4a).

**Figure 5:**
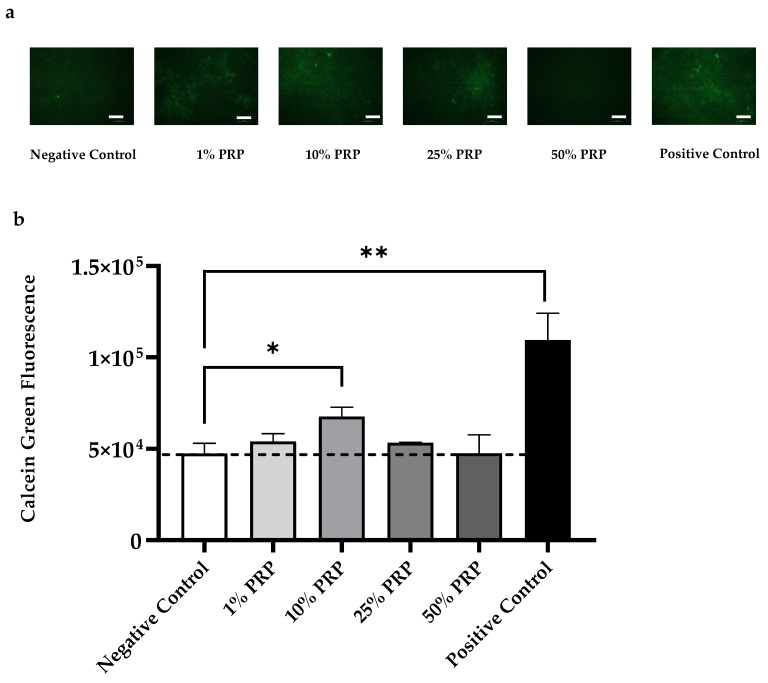
Calcein Green fluorescence of HOB-01-C1 cells treated with 1–50% PRP dilutions: (**a**) fluorescence microscopy images (10×) of PRP Sample 3 (chosen as a representative example) stained after 12 days of culture (T12) Scale bar 100 μm; (**b**) Semi-quantitative analysis, acquired at T12. Data are expressed as mean fluorescence ± SD of three independent experiments. (* *p* < 0.05; ** *p* < 0.001).

Calcein Green staining assay, performed on HOB-01-C1 cells treated with different PRP dilutions, confirmed what was previously observed with Alizarin Red S. Figure 5 reports the representative results obtained for Sample 3. The results confirm that 10% PRP led to a significantly higher deposition of calcium phosphates compared to Negative Control (* *p* < 0.05)

#### 2.4.3. Gene Expression

**Figure 6:**
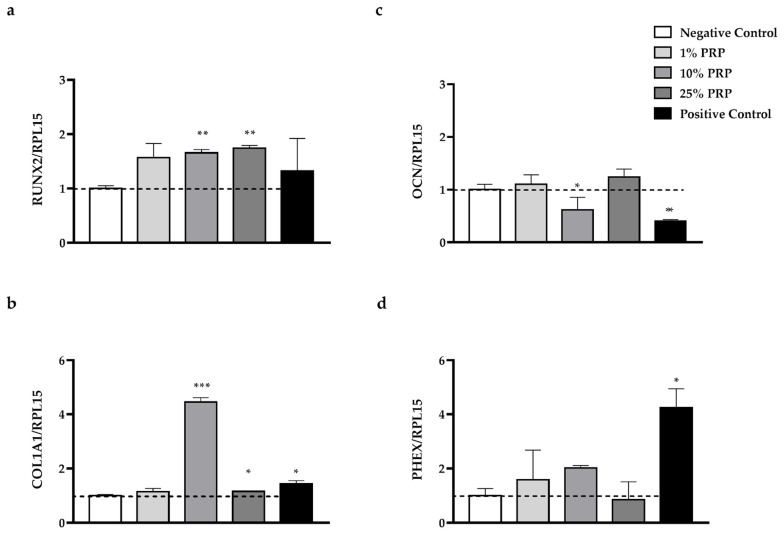
Gene expression of HOB-01-C1 cells at a day 12 after seeding (T12) and treated with different dilutions of PRP (1–25%). (**a**) RUNX2; (**b**) COL1A1; (**c**) OCN; (**d**) PHEX. Each gene was normalized for Ribosomal Protein 15 (RPL15). Data are reported as fold of expression (Fold change) vs. Negative Control and represented as mean ± SD of three independent experiments performed on sample 4. Statistical analysis was performed by one-way Anova. (* *p* < 0.05; ** *p* < 0.01; *** *p* < 0.001).

To further investigate the role of PRP in osteogenic differentiation, gene expression analysis was performed on a panel of key genes known to regulate the transition between differentiated osteoblasts and osteocytes. Time-table is reported in Figure A2. The results revealed differences in the relative expression levels of genes involved in osteogenic differentiation depending on the PRP dilution (1–25%, Figure 6).

The expression of runt-related transcription factor-2 (RUNX2, Figure 6a), which is considered the master gene of osteogenesis, increased after PRP treatment: the fold-change compared to the negative control was statistically significant after treatment with 10% and 25% dilutions (Fold change vs. Negative Control: 1% PRP: 1.56 ± 0.26, *p* > 0.05; 10% PRP 1.65 ± 0.06, ** *p* < 0.01; 25% PRP: 1.74 ± 0.05, ** *p* < 0.01; Positive Control: 1.32 ± 0.6, *p* > 0.05).

Alpha-1 type I collagen (COL1A1, Figure 6b), whose expression is partially affected by RUNX2, significantly increased in cells treated with 10% PRP (Fold change vs. Negative Control: 1% PRP: 1.15 ± 0.12, *p* > 0.05; 10% PRP: 4.45 ± 0.16, *** *p* < 0.001; 25% PRP: 1.16 ± 0.006, * *p* < 0.05; Positive Control: 1.44 ± 0.11, * *p* < 0.05). Osteocalcin (OCN, Figure 6c), a gene exclusively expressed by osteoblasts, decreased in both positive control and after 10% PRP treatment, although the latter was not significant (Fold change vs. Negative Control: 1% PRP: 1.10 ± 0.18, *p* > 0.05; 10% PRP: 0.614 ± 0.24, *p* > 0.05; 25% PRP: 1.24 ± 0.15, *p* > 0.05; Positive Control: 0.40 ± 0.03, ** *p* < 0.01). Finally, the expression of the osteocyte-specific gene PHEX (phosphate-regulating neutral endopeptidase X-linked, Figure 6d) significantly increased in cells treated with 10% PRP, as well as in the Positive Control (Fold change vs. Negative Control: 1% PRP: 1.58 ± 1.1, *p* > 0.05; 10% PRP: 2.02 ± 0.09, *p* > 0.05; 25% PRP 0.85 ± 0.66, *p* > 0.05; Positive Control: 4.25 ± 0.7, * *p* < 0.05).

Taken together these results suggest that 10% PRP is the dilution that strongly promotes the HOB-01-C1 differentiation to a more mature phenotype.

## 3. Discussion

Despite the fact that the clinical effectiveness of PRP is widely accepted in clinical settings and its bioactivity has been demonstrated in vitro by several independent groups [21,25,29], the regenerative effects of PRP on bone healing and remodeling have still not been fully elucidated [22].

In the present study, PRP obtained by apheresis was characterized in terms of the bioactive substances’ composition and osteogenic potential to support the identification of the minimum effective concentration to promote proliferation and osteogenic differentiation.

Platelet count is the key parameter routinely assessed during PRP preparation [30], which should indirectly predict the concentration of bioactive components of the blood product. The recommended PRP platelet count ranges between 0.8 × 10^6^ and 1.2 × 10^6^/µL [30]. In our PRP samples, we found 1.22 ± 0.17 × 10^6^/µL mean PLTs, with a 4.6-fold enrichment compared to whole blood count (Table 1). These data agree with the previous literature on apheresis platelet products, as well as on whole blood PRP (WB-PRP) [31,32].

Importantly, the fibrinogen concentration (Figure 1) was also similar to that already reported in the literature for WB-PRP derived by different centrifugation systems [33].

Fibrinogen contributes to forming an extracellular matrix able to sustain GFs release from α-granules of platelets over time [17,18,19]. A recent study investigated the kinetic release of different GFs present in liquid fibrinogen at different times, demonstrating that the peak of release is reached at around 7 days [34]. In addition, another recent study demonstrates that fibrinogen itself has osteoinduction potential through the activation of the SMAD1/5/8 signaling pathway which, in turn, mediates RUNX2 gene expression [35]. According to these data, controlling fibrinogen levels might have beneficial effects on molecule delivery, on which the success of a growth factor therapy depends, and it must also be taken under consideration for the development of additional strategies to mediate osteogenic differentiation.

Thirty-six proteins were assessed in our PRP samples: 28 GFs and 8 GFs receptors (Figure 2). Among GFs, the most enriched were the PDGF isoforms, EGF and, to a minor extent, VEGF-D and TGF-β isoforms. In our samples, TGF-β levels were generally low, possibly because of sample preparation and PRP activation. TGF-β plays an important role in fracture healing and regeneration [22,36,37] and, despite the fact that repeated freeze and thaw cycles should favor TGF-β and EGF enrichment [38], it has been reported that strong centrifugation (>5800 g) significantly decreases TGF-β release [39]. Nevertheless, TGF-β concentration might have been enough to induce an increase in the expression of RUNX2. TGF-β is a master regulator of both proliferation and differentiation, through RUNX2, and may exert different effects depending on the condition. Further studies are necessary to determine which is the best concentration that may favor bone regeneration [40,41]. The most abundant proteins found in our PRP samples (PDGF-AA, -AB, -BB and EGF) were also quantified through ELISA. While PDGF isoforms were all found at a ng/mL concentration, EGF content was in the pg/mL range (Table 2). EGF (alone or in combination with other growth factors) recently gained attention for its role in osteogenic differentiation due to the ability to induce the synthesis of IL-8 and BMP [16].

As expected, we found a direct correlation of PLTs count with PDGF-AA, but not for -BB and –AB isoforms. Importantly PDGF-AA was the most abundant GF found in all the PRP tested known to exert mitogen activity in osteoblastic cells [15].

Biological effects of apheresis PRP was evaluated in HOB-01-C1 cell line, which is a well-established human osteoblast cell line with known characteristics of pre-osteocyte cells and the only available pre-osteocyte human model [27,28].

To identify the PRP amount with the best capability to induce proliferation and differentiation of HOB-01-C1 cells, we evaluated four different dilutions. The treatment with 25% (*v/v*) of PRP for three days induced the highest stimulation of human HOB-01-C1 cells’ viability up to 10 days, having even better results than the positive control. This effect might be explained by the fact that plasma rich in growth factors stimulates osteoblast autocrine expression of VEGF and HGF [9], sustaining cellular viability. This evidence was observed for 3 of the 4 PRP samples tested, while PRP Sample 2 did not give positive effects on cell viability. Despite the highest content of GFs, Sample 2 had a significantly higher amount of fibrinogen compared to the others. Fibrinogen could retain a certain amount of GFs [19,20], probably limiting their bioavailability. In accordance, Sample 4, which was the one with the lowest content of fibrinogen, gave the best results in terms of cell viability.

Interestingly, we found that 50% PRP dilution has a negative impact on cell proliferation, while the treatment with 10% induces the best proliferation profile. This behavior may be attributed to an excessive release of GFs: a negative correlation between GFs and cell viability and migration of osteoblast treated with different types of PRP derivatives has already been reported [42]. In addition, growth factors can induce both anabolic and catabolic effects on cells [43] thus potentially affecting the cellular response. Another important aspect to take into account is the contribution of citrate deriving from anticoagulant ACD-A presents in the PRP units, primarily for its role as calcium chelator, which has been demonstrated to interfere with the angiogenic and regenerative proprieties of platelets [44]. On the other hand, some authors studying the specific effect of different concentrations of citrate supplementation in promoting bone regeneration, observed that exogenous supplementation of citrate is dose-dependent and that 200 μM is the optimal concentration able to induce the elevation of osteogenic markers [45]. The citrate concentrations measured in the PRP units used for this study were under 8 mM for 50% and 2 mM for 10 % dilutions, respectively (data not shown). Therefore, further studies are necessary in order to understand whether the high amount of citrate present in PRP units may be responsible for a regenerative effect.

To the best of our knowledge, this is the first in vitro study demonstrating that just 3 days of PRP treatment can induce long-term deposition (12 days) of calcium salts in preosteocytes. Our observations were confirmed by both Calcein Green Staining and Alizarin Red Assay.

To further support the osteogenic potential of PRP, we analyzed the expression of genes involved in the transition between differentiated osteoblasts and osteocytes that characterizes the process of osteogenesis [4].

RUNX2 is considered the master gene of osteogenesis, also affecting COL1A1 expression [46,47] and other osteoblast/osteocyte markers [48,49]; OCN is exclusively produced by osteoblasts, whereas PHEX is expressed only by mature osteocytes [50,51,52,53]. Gene analysis, assessed on cells treated with PRP, revealed that RUNX2 was overexpressed in all the treated samples (Figure 6). This evidence supports the hypothesis that 3 days of treatment with PRP can sustain osteogenesis over a longer period, as this transcription factor is expressed by neither pre-osteoblasts nor osteo-chondroprogenitor cells [54]. OCN is a mature osteoblast marker that influences hydroxyapatite formation [55], and whose production increases in the late stage of osteogenesis, to decrease again as the differentiation proceeds [50,51,52,53]. OCN gene appears to be underexpressed after 12 days of treatment with 10% PRP and Positive Control compared to Negative Control (Figure 6), suggesting that the cells had already reached a differentiated stage. This trend seems to be confirmed by PHEX, which is more expressed in 10% PRP and in the Positive Control compared to Negative Control. PHEX plays a role in phosphate homeostasis and mineralization of the bone matrix and is typically upregulated in pre-osteocyte/osteocyte [50,52,53]: thus, our results prove that cells treated for 3 days with 10% PRP acquire a more differentiated gene profile compared to other dilutions.

Type I collagen is one of the earliest osteoblast markers and the most abundant component of the immature bone matrix [56]. COL1A1, the gene that regulates its production, starts to be expressed in immature osteoprogenitors and lasts during the whole differentiation process, to increase in mature osteoblasts [53]. Again, COL1A1 was highly expressed particularly in cells treated with 10% PRP (Figure 6). Much evidence indicates that TGF-β and PDGF-BB are able to upregulate COL1A1 [57,58]: these GFs, present in PRP samples, at the proper concentration could stimulate greater Type I collagen production.

Taken together, our data represent a step towards the standardization and use and of apheresis PRP in bone healing and remodeling. We provided for the first time a deep characterization of this blood product, taking into consideration the interindividual variations in terms of PLTs count, GFs and fibrinogen concentrations, as well as the potential impact in regenerative medicine. The evidence collected suggest that apheresis PRP might be useful in bone healing and remodeling, and that 10% dilution is already effective in stimulating cell differentiation.

PRP obtained by apheresis can be collected in large amounts in donor centers and, compared to autologous WB-PRP, has the advantage of being technically standardized: indeed, there is a wide heterogeneity of protocols for WB-PRP preparation, and reproducibility is operator-dependent [23,24]. However, the devices and protocols used for PRP preparation are not the only factors contributing to wide disparities in biological activity; peripheral blood from an individual donor is characterized by a large inherent variability in the concentrations of platelets and GFs [59].

The efficacy of PRP could depend on several factors, such as the donor’s health status or age. Concerning WB-PRP preparation, previous studies have shown that the content of cytokines and biomolecules differ between elderly and young people [60], with aged PRP containing more pro-inflammatory cytokines than young PRP [61].

The PRP analysed in this work were obtained with identical processes, with the unique variables of donors’ sex and age. Both female donors were under the age of 40. Despite the fact that none of the samples differed for the PLTs count, the PRP from female donors contained higher GFs levels. This result is in line with that reported by Evanson et al. demonstrating higher GFs for females and for those <25 years old [62]. However, in agreement with previous studies [42,63], higher cytokines and GFs concentration seems to not be related to a positive effect on cell proliferation (see Figure 2).

Further studies on a larger sample size, evaluating the effect of donors age- and gender-related alterations in GFs concentrations in different cellular models are necessary before moving towards a standardized clinical application. Moreover, it could be of great interest to evaluate the suitability of PRP cotreatment regimen with other clinically validated methods, to seek a possible synergistic effect of PRP with other already commonly accepted clinical therapies for bone regeneration.

## 4. Materials and Methods

### 4.1. Study Population

The study was conducted by the Transfusion Medicine Unit of the Azienda USL-IRCCS di Reggio Emilia and approved by the Institutional Board Review on 10 January 2019 (Reggio Emilia Ethics Committee, protocol number 2019/0003319).

Leukodepleted platelet-rich plasma (PRP) was collected by apheresis from four donors using an automated blood collection system (Mobile Collection System MCS+, Haemonetics Corp., Boston, MA, USA), according to the manufacturer’s instructions. Acid Citrate Dextrose Solution A (ACD-A) was used as anticoagulant. The following information was collected for each donor: sex, age, weight, blood group, blood pressure, complete blood count. All donors were not taking antiplatelet or anticoagulant drugs within 1 week before donation. All donors signed informed consent according to the Declaration of Helsinki.

### 4.2. PRP Samples Collection

The four PRP units collected were irradiated and left in agitation for 2 days after donation at room temperature. Platelet count on PRP units was performed immediately after irradiation using CELL-DYN Ruby Hematology Analyzer (Abbott, Chicago, IL, USA). Subsequently, PRP units were divided into aliquots and stored at—80 °C. Platelet activation was carried out with a single freeze–thaw cycle before being tested on cell cultures, and a double freeze–thaw cycle for biochemical analysis. EDTA-free Protease Inhibitor Cocktail (ab201111, Abcam, Cambridge, UK) was added to the PRP aliquots destined for biochemical analysis before the storage, to avoid protein degradation.

### 4.3. Human Growth Factor Antibody Array

The relative content of 36 GFs was assessed on 10-fold diluted PRP samples using the Growth Factor Human Membrane Antibody Array (ab134002, Abcam, Cambridge, UK), according to the manufacturer’s instructions. A complete release of GFs from platelets was achieved after two freeze–thaw cycles and hard spinning (15.000 rpm for 5 min). Chemiluminescence signals on the membranes were acquired with iBright^TM^ CL1500 Imaging System (Invitrogen™) and semi-quantitative analysis was performed with iBright Analysis Software (Invitrogen™). GFs relative expressions on different membranes were normalized to a control array, chosen as control spot Sample 1.

### 4.4. Enzyme-Linked Immunosorbent Assay (ELISA)

Fibrinogen (FIB) concentration (μg/mL) was quantified with Human Fibrinogen ELISA Kit (ab108842, Abcam, Cambridge, UK), following the manufacturer’s instructions. Each measure was performed on three different PRP replicates of the same unit and assessed in triplicate. GFs (Table 3) concentration was assessed by the following Human ELISA kits (Sigma-Aldrich s.r.l., Milan): PDGF-AA (RAB0394), PDGF-BB (RAB0397), PDGF-AB (RAB0396), EGF (RAB0149). Each measure was performed on four different PRP dilutions, except for the PDGF-AA (three dilutions), and assessed in duplicate. ELISA 96-well plates were read with Glomax^®^ Discover Microplate Reader (Promega, Madison, WI, USA).

### 4.5. Cell Culture

Human HOB-01-C1 cells [64] were cultured in different conditions to evaluate cell viability and osteogenic potential of platelet-rich plasma. PRP samples from different donors (Sample 1, Sample 2, Sample 3, Sample 4) were diluted with culture medium to obtain 1%, 10%, 25%, or 50% volume/volume working dilutions for cell cultures.

### 4.6. Resazurin Assay

HOB-01-C1 cells were seeded at a density of 500 cells/well in 96-well plates (EUROCLONE SpA, Milan, Italy) in Dulbecco’s Modified Eagle Medium (DMEM) Low Glucose (EUROCLONE SpA, Milan, Italy) supplemented with 1% fetal bovine serum (EUROCLONE SpA, Milan, Italy), 200 mM Glutamine and 1% Streptomycin/Penicillin (both from Sigma-Aldrich s.r.l., Milan, Italy). Cells were seeded 24 h prior experiment incubated at 37 °C, 5% CO_2_. The day after, culture medium was changed with Dulbecco’s Modified Eagle Medium Low Glucose supplemented 200 mM Glutamine and 1%, Streptomycin/Penicillin without serum supplement and treated with different percentages of apheresis freeze thawed PRP (1%; 10%; 25%; 50%) from a single donor for 3 days. After 3 days of treatment, medium containing PRP was replaced with the same medium previously described, supplemented with 0.1% Human Serum AB (EUROCLONE SpA, Milan, Italy) without PRP. Culture medium was changed every two or three days until day 10 (T10). Positive control samples and negative control samples were cultured in growth medium containing 10% or 0.1% of Human AB Serum, respectively, without PRP, replaced every two or three days with the same growth medium. Each condition was performed in 4 replicates. A 10X Resazurin stock solution (Sigma-Aldrich s.r.l., Milan, Italy) was prepared in sterile Phosphate Buffered Saline (EUROCLONE SpA, Milan, Italy). The 1X working solution was obtained by diluting the stock solution in DMEM Low Glucose. The Resazurin Cell Viability Assay was performed before the treatment (T0) and at T3, T5, T7, T10. Growth medium was removed, and cells were rinsed with sterile PBS, then incubated with 120 μL of Resazurin working solution in each well for 1 h at 37 °C to allow Resazurin’s reduction in Resorufin. Relative fluorescent units (RFU) were acquired on 100 μL samples by EnSpire^®^ Multimode Plate Reader (Perkin Elmer, Waltham, MA, USA).

### 4.7. Alizarin Red Assay

To evaluate the osteogenic potential of Platelet Rich Plasma, HOB-01-C1 cells were seeded at a density of 50.000/cm^2^ in 24 well plates (Falcon^®^) in DMEM Low Glucose supplemented with 1% fetal bovine serum (EUROCLONE SpA, Milan, Italy), 200 mM Glutamine and 1% Streptomycin/Penicillin. Cells were allowed to adhere for 24 h and incubated at 37 °C with 5% CO_2_. The day after, the culture medium was replaced with an osteogenic medium composed as follows: MEM ALPHA MEDIUM (EUROCLONE SpA, Milan, Italy) supplemented with 0.1% Human Serum AB (EUROCLONE SpA, Milan, Italy), 200 mM Glutamine, 1%, Streptomycin/Penicillin, 100 μM ascorbic acid, 2.5 mM β-glycerolphosphate and 40 μg/mL L-proline. Cells were treated with different percentages of freeze thawed PRP (1%; 10%; 25%; 50%) for 3 days. Osteogenic medium containing different percentages of PRP was removed after 3 days and then replaced every two or three days until day 14 (T14). The controls were not treated with PRP and cultured in osteogenic medium containing 0.1% of Human AB Serum (Negative Control) and 10% of Human AB Serum (Positive Control), replaced every two or three days with the same osteogenic medium. Each condition was performed in triplicate. To evaluate calcium deposition, cells were rinsed with PBS and fixed in 4% buffered formaldehyde (Sigma-Aldrich s.r.l., Milan, Italy) solution. Alizarin Red S Staining Quantification Assay (ScienCell^TM^, San Diego, CA, USA) was performed according to the manufacturer’s instructions. Absorbance at 405 nm was measured by EnSpire^®^ Multimode Plate Reader.

### 4.8. Calcein Green Staining

Cells were treated with osteogenic medium plus different percentages of freeze thawed PRP (1%; 10%; 25%; 50%) for 3 days. PRP was removed after 3 days and then replaced every two or three days until day 12 (T12). The controls were not treated with PRP and cultured in osteogenic medium containing 0.1% of Human AB Serum (Negative Control) and 10% of Human AB Serum (Positive Control), replaced every two or three days with the same osteogenic medium. Each condition was performed in triplicate, Calcein Green (ex/em = 495/515 nm) was added to the osteogenic medium 24 hours prior the detection by reading the 24-well plate at 512 nm with EnSpire® Multimode Plate Reader.

### 4.9. Gene Expression Analysis

Total RNA was extracted at day 12 (T12) by PureLink^TM^ RNA Mini kit (Invitrogen, Thermo Fisher Scientific, Inc., Waltham, MA, USA); 1 μg of RNA/sample was reverse transcribed to cDNA with RevertAid RT Reverse Transcription Kit (Thermo Fisher Scientific Inc., Waltham, MA, USA) following the manufacturer’s instructions. cDNA samples were then diluted with nuclease-free water up to a total volume of 200 μL. Real-Time PCR mix was composed of 3 μL of cDNA, 3.44 μL of molecular biology grade water (EUROCLONE SpA, Milan, Italy), 7 μL of Luna^®^ Universal qPCR Master Mix (New England BioLabs Inc., Ipswich, MA, USA) and 0.56 μL of primer, for a final reaction volume of 14 μL. PCR was performed in a 36 well Rotor-Gene 3000 (Rotor-Gene™ 3000, version 5.0.60, Mortlake, Australia). The relative expressions of genes typically involved in osteogenesis (RUNX2, OCN, COL1A1, PHEX) were normalized for the housekeeping gene RPL15. Relative changes in expression were analyzed with REST2009 (QIAGEN, Hiden, Germany), software that uses Pfaffl method (see Table 4) [65]. Technical condition for gene expression analysis on the selected genes has been determined as previously reported [66,67,68].

### 4.10. Statistical Analysis

Viability curves were analyzed using 4PL sigmoidal curve. Data obtained by Growth Factors Array were normalized and after product characterization and analyzed with two-way ANOVA. Data obtained by ELISA and evaluation of osteogenic potential data (Alizarin Red S Quantification, Calcein Green Staining, gene expression analysis) were analyzed by One-Way ANOVA. Kruskas-Wallis test was used as non-parametric test for EGF values of ELISA. Graphs were obtained using GraphPad Prism 8.0 software (GraphPad Software, La Jolla, CA; USA). Data are shown as mean values ± SD. Asterisks indicate statistical significance (* *p* < 0.05; ** *p* < 0.01; *** *p* < 0.001). Spearman’s nonparametric correlation test has been used to identify positive or negative correlations between PLTs count and GFs amount released by PRP. 

## Figures and Tables

**Table 1:** PLTs count on donors’ whole blood and PRP units.

Sample	PLTs Recovery (10^3^/µL)		Enrichment
	Whole Blood	PRP	
1	300	992	3.3
2	256	1342	5.4
3	252	1362	5.4
4	249	1197	4.8

**Table 2:** PDGF (isoforms AA, BB, AB) and EGF concentrations (ng/mL) in the PRP samples, number of replicates = 4. Values are reported as mean values ± SD (* *p* < 0.05, ** *p* < 0.01, *** *p* < 0.001).

	Growth Factors (ng/mL)			
PRP	PDGF-AA	PDGF-AB	PDGF-BB	EGF
**1**	777.25 ± 147.07	67.82 ± 2.16	72.29 ± 4.98	0.65 ± 0.05 *
**2**	1694.25 ± 311.23 **	72.10 ± 2.53 ***	98.10 ± 12.13	0.62 ± 0.13
**3**	1030.07± 62.74	55.58 ± 5.26	83.18 ± 12.26	0.52 ± 0.03
**4**	1046.06 ± 176.25	53.10 ± 3.42	46.26 ± 8.18 ***	0.48 ± 0.37

**Table 3:** PRP dilution in the culture medium, and corresponding GFs concentration, used to treat HOB-01-C1 cells. Data are expressed as percentage volume in the culture medium for PRP dilution and mean ± SD for GFs concentration.

	GFs (ng/mL)			
Dilution	PDGF-AA	PDGF-AB	PDGF-BB	EGF
1%	11.36 ± 3.9	0.61 ± 0.1	0.74 ± 0.2	5.65 ± 0.82 (pg/mL)
10%	113.67 ± 39	6.17 ± 0.9	7.45 ± 2.2	0.056 ± 0.0
25%	284.18 ± 97	15.43 ± 2.3	18.62 ± 5.4	0.14 ± 0.02
50%	568.37 ± 195.0	30.87 ± 4.6	37.25 ± 10.9	0.28 ± 0.04

**Table 4:** Characteristics of the primers used for real-time PCR.

Gene Name	Gene Bank Number	Forward Primer	Reverse Primer	Efficiency	Product Size (bp)	Annealing Temperature (°C)
COL1A1	NM_000088	GTCGAGGGCCAAGACGAAG	CAGATCACGTCATCGCACAAC	1.07	143	59
OCN	NM_199173	TCACACTCCTCGCCCTAT	GTCAGCCAACTCGTCACA	1.12	245	60
PHEX	NM_000444	AGGCATCACATTCACCAACAAC	GCACCATTGACCCTAAACTGAG	0.82	138	59
RPL15	NM_002948	GCAGCCATCAGGTAAGCCAAG	AGCGGACCCTCAGAAGAAAGC	0.94	100	59
RUNX2	NM_004348	CCAGGCAGGCACAGTCTTC	GTCAGAGGTGGCAGTGTCATC	1.13	182	59

## Data Availability

The data presented in this study are available on request from the corresponding author. The data are not publicly available due to legal restrictions.

## Abbreviations

VEGF	Vascular endothelial growth factor
PDGF	Platelet-derived growth factor
TGF-β	Transforming growth factor β
EGF	Epidermal growth factor
IGF	Insulinlike growth factor
PRP	Platelet-Rich Plasma
GFs	Growth Factors
WB-PRP	Whole Blood PRP
RUNX2	Runt-related transcription factor 2
COL1A1	alpha-1 type I collagen
OCN	Osteocalcin
PHEX	Phosphate-regulating neutral endopeptidase, X-linked
RBCs	Red blood cells
PLTs	Platelets
GFRs	Growth Factors Receptors
MCSFR	Macrophage colony-stimulating factor receptor
SCSFR	Stem Cell Factor Receptor
HSAB	Human Serum AB
HGF	Hepatocyte growth factor
ACD-A	Acid Citrate Dextrose Solution A
DMEM	Dulbecco’s Modified Eagle Medium
FBS	Fetal Bovine Serum
PBS	Phosphate-buffered saline

## Appendix A

**Table A1:** Study population characteristics and procedure information.

Study Population	1	2	3	4
Sex	F	F	M	M
**Characteristics**				
Age (years)	21	38	59	56
Systolic blood pressure	115	110	120	140
Diastolic blood pressure	75	70	80	90
Weight (Kg)	78	65	75	87
WBC (10^3^/µL)	5.7	4.3	5.8	4.8
RBC (10^6^/µL)	4.8	4.8	5.1	5.6
MCV (fL)	84.1	83.5	85.2	87.2
MCH (pg)	26.9	26.3	30.1	29.2
MCHC (g/dL)	32	31.4	35.3	33.5
Haematocrit (HCT, %)	40.1	40.0	43.4	48.6
Haemoglobin (g/dL)	12.8	12.6	15.3	16.3
Platelets (10^3^ µL)	300	256	252	249
MPV (fL)	7.9	7.8	6.4	7.9
Apheresis procedure				
Total volume of processed blood (mL)	1933	1879	2260	2068
Number of cycles	4	4	5	5
ACD-A volume (mL)	258	252	297	273

## References

[1] Oryan A., Alidadi S., Moshiri A., Maffulli N. (2014). Bone regenerative medicine: Classic options, novel strategies, and future directions. J. Orthop. Surg. Res..

[2] Bigham-Sadegh A., Oryan A. (2015). Basic concepts regarding fracture healing and the current options and future directions in managing bone fractures. Int. Wound J..

[3] Bodine P.V., Komm B.S. (2002). Tissue culture models for studies of hormone and vitamin action in bone cells. Vitam. Horm..

[4] Schaffler M.B., Cheung W.Y., Majeska R., Kennedy O. (2014). Osteocytes: Master orchestrators of bone. Calcif. Tissue Int..

[5] Pittenger M.F., Mackay A.M., Beck S.C., Jaiswal R.K., Douglas R., Mosca J.D., Moorman M.A., Simonetti D.W., Craig S., Marshak D.R. (1999). Multilineage potential of adult human mesenchymal stem cells. Science.

[6] Oryan A., Alidadi S., Moshiri A., Bigham-Sadegh A. (2014). Bone morphogenetic proteins: A powerful osteoinductive compound with non-negligible side effects and limitations. Biofactors.

[7] Lieberman J.R., Daluiski A., Einhorn T.A. (2002). The role of growth factors in the repair of bone. Biology and clinical applications. J. Bone Jt. Surg. Am..

[8] Anitua E., Tejero R., Zalduendo M.M., Orive G. (2013). Plasma rich in growth factors promotes bone tissue regeneration by stimulating proliferation, migration, and autocrine secretion in primary human osteoblasts. J. Periodontol..

[9] Kim S.J., Shin Y.W., Yang K.H., Kim S.B., Yoo M.J., Han S.K., Im S.A., Won Y.D., Sung Y.B., Jeon T.S. (2009). A multi-center, randomized, clinical study to compare the effect and safety of autologous cultured osteoblast (Ossron) injection to treat fractures. BMC Musculoskelet. Disord..

[10] Mahmoud N.S., Mohamed M.R., Ali M.A.M., Aglan H.A., Amr K.S., Ahmed H.H. (2020). Osteoblast-based therapy-A new approach for bone repair in osteoporosis: Pre-clinicals. Tissue Eng. Regen. Med..

[11] Noh J.Y., Yang Y., Jung H. (2020). Molecular mechanisms and emerging therapeutics for osteoporosis. Int. J. Mol. Sci..

[12] Mokhtari H., Montaseri A., Mojaddadi A., Zonouzi H.R., Karimiyan N., Arami S. (2018). Effect of Platelet-Rich Plasma on differentiation of osteoblasts and osteoclasts in the presence of three-dimensional scaffold. Pharm. Sci..

[13] Li F.X., Li Y., Qiao C.W., Zhu J., Chen J., Zhang P.Y. (2017). Topical use of platelet-rich plasma can improve the clinical outcomes after total knee arthroplasty: A systematic review and meta-analysis of 1316 patients. Int. J. Surg..

[14] Steller D., Herbst N., Pries R., Juhl D., Hakim S.G. (2019). Positive impact of Platelet-rich plasma and Platelet-rich fibrin on viability, migration and proliferation of osteoblasts and fibroblasts treated with zoledronic acid. Sci. Rep..

[15] Colciago A., Celotti F., Casati L., Giancola R., Castano S.M., Antonini G., Sacchi M.C., Negri-Cesi P. (2009). In Vitro Effects of PDGF Isoforms (AA, BB, AB and CC) on migration and proliferation of SaOS-2 osteoblasts and on migration of human osteoblasts. Int. J. Biomed. Sci..

[16] Del Angel-Mosqueda C., Gutiérrez-Puente Y., López-Lozano A.P., Romero-Zavaleta R.E., Mendiola-Jiménez A., Medina-De la Garza C.E., Márquez-M M., de la Garza-Ramos M.A. (2015). Epidermal growth factor enhances osteogenic differentiation of dental pulp stem cells in vitro. Head Face Med..

[17] He L., Lin Y., Hu X., Zhang Y., Wu H. (2009). A comparative study of platelet-rich fibrin (PRF) and platelet-rich plasma (PRP) on the effect of proliferation and differentiation of rat osteoblasts in vitro. Oral Surg. Oral Med. Oral Pathol. Oral Radiol. Endod..

[18] Zumstein M.A., Berger S., Schober M., Boileau P., Nyffeler R.W., Horn M., Dahinden C.A. (2012). Leukocyte- and platelet-rich fibrin (L-PRF) for long-term delivery of growth factor in rotator cuff repair: Review, preliminary results and future directions. Curr. Pharm. Biotechnol..

[19] Sitek P., Wysocka-Wycisk A., Kępski F., Król D., Bursig H., Dyląg S. (2013). PRP-fibrinogen gel-like chondrocyte carrier stabilized by TXA-preliminary study. Cell Tissue Bank..

[20] Martino M.M., Briquez P.S., Ranga A., Lutolf M.P., Hubbell J.A. (2013). Heparin-binding domain of fibrin(ogen) binds growth factors and promotes tissue repair when incorporated within a synthetic matrix. Proc. Natl. Acad. Sci. USA.

[21] Graziani F., Ivanovski S., Cei S., Ducci F., Tonetti M., Gabriele M. (2006). The in vitro effect of different PRPconcentrations on osteoblasts and fibroblasts. Clin. Oral Implant. Res..

[22] Oryan A., Alidadi S., Moshiri A. (2016). Platelet-rich plasma for bone healing and regeneration. Expert Opin. Biol. Ther..

[23] Durante C., Agostini F., Abbruzzese L., Toffola R.T., Zanolin S., Suine C., Mazzucato M. (2013). Growth factor release from platelet concentrates: Analytic quantification and characterization for clinical applications. Vox Sang..

[24] Agostini F., Polesel J., Battiston M., Lombardi E., Zanolin S., da Ponte A., Astori G., Durante C., Mazzucato M. (2017). Standardization of platelet release products for clinical applications in cell therapy: A mathematical approach. J. Transl. Med..

[25] Camargo P.M., Lekovic V., Weinlaender M., Vasilic N., Madzarevic M., Kenney E.B. (2002). Platelet-rich plasma and bovine porous bone mineral combined with guided tissue regeneration in the treatment of intrabony defects in humans. J. Periodontal. Res..

[26] Dallari D., Savarino L., Stagni C., Cenni E., Cenacchi A., Fornasari P.M., Albisinni U., Rimondi E., Baldini N., Giunti A. (2007). Enhanced tibial osteotomy healing with use of bone grafts supplemented with platelet gel or platelet gel and bone marrow stromal cells. J. Bone Jt. Surg. Am..

[27] Divieti Pajevic P. (2020). New and Old Osteocytic Cell Lines and 3D Models. Curr. Osteoporos. Rep..

[28] Billiard J., Moran R.A., Whitley M.Z., Chatterjee-Kishore M., Gillis K., Brown E.L., Komm B.S., Bodine P.V.N. (2003). Transcriptional profiling of human osteoblast differentiation. J. Cell. Biochem..

[29] Gentile P., Garcovich S. (2020). Systematic Review—The potential implications of different Platelet-Rich-Plasma (PRP) concentrations in regenerative medicine for tissue repair. Int. J. Mol. Sci..

[30] Aprili G., Gandini G., Guaschino R., Mazzucco L., Salvaneschi L., Vaglio S. (2013). SIMTI Working Group. SIMTI recommendations on blood components for non-transfusional use. Blood Transfus..

[31] Singh R.P., Marwaha N., Malhotra P., Dash S. (2009). Quality assessment of platelet concentrates prepared by platelet rich plasma-platelet concentrate, buffy coat poor-platelet concentrate (BC-PC) and apheresis-PC methods. Asian J. Transfus. Sci..

[32] Marck R.E., Gardien K.L.M., Vlig M., Breederveld R.S., Middelkoop E. (2019). Growth factor quantification of Platelet-Rich Plasma in burn patients compared to matched healthy volunteers. Int. J. Mol. Sci..

[33] Castillo T.N., Pouliot M., Kim H.J., Dragoo J.L. (2011). Comparison of growth factor and platelet concentration from commercial platelet-rich plasma separation systems. Am. J. Sports Med..

[34] Serafini G., Lopreiato M., Lollobrigida M., Lamazza L., Mazzucchi G., Fortunato L., Mariano A., Scotto d’Abusco A., Fontana M., De Biase A. (2020). Platelet Rich Fibrin (PRF) and Its Related Products: Biomolecular Characterization of the Liquid Fibrinogen. J. Clin. Med..

[35] Kidwai F., Edwards J., Zou L., Kaufman D.S. (2016). Fibrinogen Induces RUNX2 Activity and Osteogenic Development from Human Pluripotent Stem Cells. Stem Cells.

[36] Krüger J.P., Freymannx U., Vetterlein S., Neumann K., Endres M., Kaps C. (2013). Bioactive factors in platelet-rich plasma obtained by apheresis. Transfus Med. Hemother..

[37] ten Dijke P., Fu J., Schaap P., Roelen B.A. (2003). Signal transduction of bone morphogenetic proteins in osteoblast differentiation. J. Bone Jt. Surg. Am..

[38] Strandberg G., Sellberg F., Sommar P., Ronaghi M., Lubenow N., Knutson F., Berglund D. (2017). Standardizing the freeze-thaw preparation of growth factors from platelet lysate. Transfusion.

[39] Rodriguez A.E., Gisbert S., Palazón A., Alio J.L. (2020). Quantification of growth factors and fibronectin in diverse preparations of Platelet-Rich Plasma for the treatment of ocular surface disorders (E-PRP). Transl. Vis. Sci. Technol..

[40] Alliston T., Choy L., Ducy P., Karsenty G., Derynck R. (2001). TGF-β-induced repression of CBFA1 by Smad3 decreases cbfa1 and osteocalcin expression and inhibits osteoblast differentiation. EMBO J..

[41] Lee K.-S., Kim H.-J., Li Q.-L., Chi X.-Z., Ueta C., Komori T., Wozney J.M., Kim E.-G., Choi J.-Y., Ryoo H.-M. (2000). Runx2 is a common target of transforming growth factor β1 and bone morphogenetic protein 2, and cooperation between Runx2 and Smad5 induces osteoblast-specific gene expression in the pluripotent mesenchymal precursor cell line C2C12. Mol. Cell. Biol..

[42] Fernández-Medina T., Vaquette C., Ivanovski S. (2019). Systematic comparison of the effect of four clinical-grade platelet rich hemoderivatives on osteoblast behaviour. Int. J. Mol. Sci..

[43] Schmidt M.B., Chen E.H., Lynch S.E. (2006). A review of the e_ects of insulin-like growth factor and platelet derived growth factor on in vivo cartilage healing and repair. Osteoarthr. Cartil..

[44] Oneto P., Zubiry P.R., Schattner M., Etulain J. (2020). Anticoagulants interfere with the angiogenic and regenerative responses mediated by platelets. Front. Bioeng. Biotechnol..

[45] Ma C., Tian X., Kim J.P., Xie D., Ao X., Shan D., Lin Q., Hudock M.R., Bai X., Yang J. (2018). Citrate-based materials fuel human stem cells by metabonegenic regulation. PNAS.

[46] Schroeder T.M., Jensen E.D., Westendorf J.J. (2005). Runx2: A master organizer of gene transcription in developing and maturing osteoblasts. Birth Defects Res. C Embryo Today.

[47] Cazzaniga A., Maier J.A.M., Castiglioni S. (2016). Impact of simulated microgravity on human bone stem 502 cells: New hints for space medicine. Biochem. Biophys. Res. Commun..

[48] Nakashima K., de Crombrugghe B. (2003). Transcriptional mechanisms in osteoblast differentiation and bone formation. Trends Genet..

[49] Kobayashi T., Kronenberg H. (2005). Minireview: Transcriptional regulation in development of bone. Endocrinology.

[50] Dallas S.L., Prideaux M., Bonewald L.F. (2013). The osteocyte: An endocrine cell ... and more. Endocr. Rev..

[51] Wang L., Li Z.Y., Wang Y.P., Wu Z.H., Yu B. (2015). Dynamic expression profiles of marker genes in osteogenic differentiation of human bone marrow-derived mesenchymal stem cells. Chin. Med. Sci. J..

[52] Westbroek I., De Rooij K.E., Nijweide P.J. (2002). Osteocyte-specific monoclonal antibody MAb OB7.3 is directed against Phex protein. J. Bone Miner. Res..

[53] Aubin J.E. (2001). Regulation of osteoblast formation and function. Rev. Endocr. Metab. Disord..

[54] Franz-Odendaal T.A., Hall B.K., Witten P.E. (2006). Buried alive: How osteoblasts become osteocytes. Dev. Dyn..

[55] Tsao Y.T., Huang Y.J., Wu H.H., Liu Y.A., Liu Y.S., Lee O.K. (2017). Osteocalcin Mediates Biomineralization during Osteogenic Maturation in Human Mesenchymal Stromal Cells. Int. J. Mol. Sci..

[56] Fujisawa R., Tamura M. (2012). Acidic bone matrix proteins and their roles in calcification. Front. Biosci. (Landmark Ed.).

[57] Zhang Y., Wang Y., Shi B., Cheng X. (2007). A platelet-derived growth factor releasing chitosan/coral composite scaffold for periodontal tissue engineering. Biomaterials.

[58] Glueck M., Gardner O., Czekanska E., Alini M., Stoddart M.J., Salzmann G.M., Schmal H. (2015). Induction of osteogenic differentiation in human mesenchymal stem cells by crosstalk with osteoblasts. BioRes. Open Access.

[59] Cho H.S., Song I.H., Park S.Y., Sung M.C., Ahn M.W., Song K.E. (2011). Individual variation in growth factor concentrations in platelet-rich plasma and its influence on human mesenchymal stem cells. Korean J. Lab. Med..

[60] Larsson A., Carlsson L., Gordh T., Lind A.-L., Thulin M., Kamali-Moghaddam M. (2015). The effects of age and gender on plasma levels of 63 cytokines. J. Immunol. Methods.

[61] O’Donnell C., Migliore E., Grandi F.C., Koltsov J., Lingampalli N., Cisar C., Indelli P.F., Sebastiano V., Robinson W.H., Bhutani N. (2019). Platelet-rich plasma (PRP) from older males with knee osteoarthritis depresses chondrocyte metabolism and upregulates inflammation. J. Orthop. Res..

[62] Evanson J.R., Guyton M.K., Oliver D.L., Hire J.M., Topolski R.L., Zumbrun S.D., McPherson J.C., Bojescul J.A. (2014). Gender and age differences in growth factor concentrations from platelet-rich plasma in adults. Mil. Med..

[63] Delgado D., Bilbao A.M., Beitia M., Garate A., Sánchez P., González-Burguera I., Isasti A., López De Jesús M., Zuazo-Ibarra J., Montilla A. (2021). Effects of Platelet-Rich Plasma on Cellular Populations of the Central Nervous System: The Influence of Donor Age. Int. J. Mol. Sci..

[64] Bodine P.V., Vernon S.K., Komm B.S. (1996). Establishment and hormonal regulation of a conditionally transformed preosteocytic cell line from adult human bone. Endocrinology.

[65] Pfaffl M.W. (2001). A new mathematical model for relative quantification in real-time RT-PCR. Nucleic Acids Res..

[66] Toscani D., Palumbo C., Dalla Palma B., Ferretti M., Bolzoni M., Marchica V., Sena P., Martella E., Mancini C., Ferri V. (2016). The proteasome inhibitor bortezomib maintains osteocyte viability in multiple myeloma patients by reducing both apoptosis and autophagy: A new function for proteasome inhibitors. J. Bone Miner. Res..

[67] Giuliani N., Colla S., Morandi F., Lazzaretti M., Sala R., Bonomini S., Grano M., Colucci S., Svaldi M., Rizzoli V. (2005). Myeloma cells block RUNX2/CBFA1 activity in human bone marrow osteoblast progenitors and inhibit osteoblast formation and differentiation. Blood.

[68] Negri F., Bozzetti C., Pedrazzi G., Azzoni C., Bottarelli L., Squadrilli A., Lagrasta C., Tamagnini I., Bisagni A., Ragazzi M. (2019). High levels of Notch intracellular cleaved domain are associated with stemness and reduced bevacizumab efficacy in patients with advanced colon cancer. Oncol. Rep..

